# Algorithms for the Capture and Adjudication of Prevalent and Incident Diabetes in UK Biobank

**DOI:** 10.1371/journal.pone.0162388

**Published:** 2016-09-15

**Authors:** Sophie V Eastwood, Rohini Mathur, Mark Atkinson, Sinead Brophy, Cathie Sudlow, Robin Flaig, Simon de Lusignan, Naomi Allen, Nishi Chaturvedi

**Affiliations:** 1 Institute of Cardiovascular Sciences, University College London, London, United Kingdom; 2 Department of Non-Communicable Diseases, London School of Hygiene & Tropical Medicine, London, United Kingdom; 3 CIPHER (Centre for the Improvement of Population Health through e-Records Research) College of Medicine, Swansea University, Swansea, United Kingdom; 4 Centre for Clinical Brain Sciences (CCBS), University of Edinburgh, Edinburgh, United Kingdom; 5 Department of Clinical and Experimental Medicine, University of Surrey, Guilford, United Kingdom; 6 Nuffield Department of Population Health, University of Oxford, Oxford, United Kingdom; 7 United Kingdom, Biobank, Stockport, United Kingdom; Deutsches Diabetes-Zentrum Leibniz-Zentrum fur Diabetes-Forschung, GERMANY

## Abstract

**Objectives:**

UK Biobank is a UK-wide cohort of 502,655 people aged 40–69, recruited from National Health Service registrants between 2006–10, with healthcare data linkage. Type 2 diabetes is a key exposure and outcome. We developed algorithms to define prevalent and incident diabetes for UK Biobank. The algorithms will be implemented by UK Biobank and their results made available to researchers on request.

**Methods:**

We used UK Biobank self-reported medical history and medication to assign prevalent diabetes and type, and tested this against linked primary and secondary care data in Welsh UK Biobank participants. Additionally, we derived and tested algorithms for incident diabetes using linked primary and secondary care data in the English Clinical Practice Research Datalink, and ran these on secondary care data in UK Biobank.

**Results and Significance:**

For prevalent diabetes, 0.001% and 0.002% of people classified as “diabetes unlikely” in UK Biobank had evidence of diabetes in their primary or secondary care record respectively. Of those classified as “probable” type 2 diabetes, 75% and 96% had specific type 2 diabetes codes in their primary and secondary care records. For incidence, 95% of people with the type 2 diabetes-specific C10F Read code in primary care had corroborative evidence of diabetes from medications, blood testing or diabetes specific process of care codes. Only 41% of people identified with type 2 diabetes in primary care had secondary care evidence of type 2 diabetes. In contrast, of incident cases using ICD-10 type 2 diabetes specific codes in secondary care, 77% had corroborative evidence of diabetes in primary care. We suggest our definition of prevalent diabetes from UK Biobank baseline data has external validity, and recommend that specific primary care Read codes should be used for incident diabetes to ensure precision. Secondary care data should be used for incident diabetes with caution, as around half of all cases are missed, and a quarter have no corroborative evidence of diabetes in primary care.

## Introduction

UK Biobank (UKB) is a prospective cohort comprising half a million participants recruited from the general population aged 40–69 years, with genotypic, phenotypic and linked health care record data. It was designed to improve prevention, diagnosis and treatment of diseases of middle and old age[1]. Diabetes is one of the most prevalent conditions in the UKB population, with around twenty-five thousand cases self-reported at baseline[2], and is forecast to be the most common disease outcome, with an estimated 25,000 incident cases by 2017, and 40,000 by 2022[3]. Thus, diabetes forms a key exposure, effect modifier and outcome in UKB; its accurate ascertainment and sub-classification is a priority in ensuring the usefulness of UKB data to the research community. The UKB Diabetes Outcomes Adjudication Group was convened to provide guidance in defining prevalent and incident diabetes diagnoses within UKB, using questionnaire, linked primary and secondary care data.

There is no single agreed gold standard for establishing prevalent diabetes in observational studies. Validity of diabetes self-report in epidemiological studies varies considerably, with positive predictive values ranging between 67–92%[4–6]. Discrepancies are also observed when self-completed versus interviewer-delivered questionnaire are compared, with a reported 74% concordance between these two methods[7]. UK studies examining the accuracy of diabetes diagnosis in primary care records have consistently identified incorrectly diagnosed diabetes, misclassification of diabetes type, and use of ambiguous Read codes (5–17%, 10–26% and 9–15% respectively)[8,9]. Reliance on secondary care data alone will miss a significant proportion of type 2 diabetes cases, as it is largely managed in primary care.

We aimed to develop algorithms to establish prevalent and incident diabetes diagnoses in UK Biobank, using self-report and primary and secondary care data. Additionally, we offer guidance on their usage for researchers. Algorithms will be implemented by UKB and their results made available to researchers on request.

## Methods

**Fig 1:**
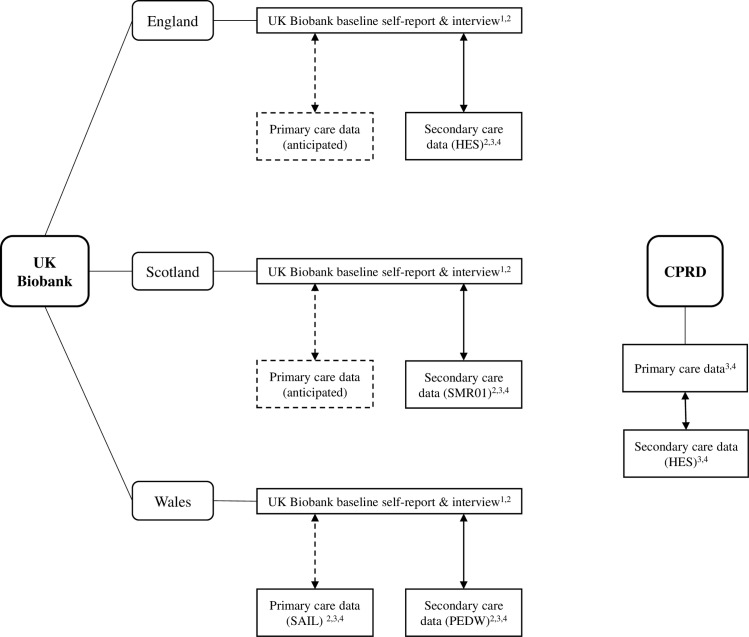
Data sources used in the development of UK Biobank diabetes prevalence and incidence algorithms. Solid arrows indicate established linkages, dotted arrows indicate anticipated linkages. ^1^data used to derive prevalence algorithms, ^2^data used to test prevalence algorithms, ^3^data used to derive incidence algorithms, ^4^data used to test incidence algorithms. HES = Hospital episode statistics, SMR01 = Scottish morbidity record, SAIL = Secure anonymised information linkage databank, PEDW = patient episode database for Wales, CPRD = clinical practice research datalink.

Three data sources were used in these analyses (Fig 1).

Firstly, baseline data and linked hospital admissions records dating from 1997 to 2012 (with complete data to 2010) were requested from the UK Biobank (UKB) data repository (n = 502,665). UKB participants were recruited from NHS registers and assessed at 22 centres between 2006–2010 across the UK[3]. Approximately 9 million invitation letters were sent to yield half a million participants, a response rate of 5.5%[10]. Baseline assessment included a health and lifestyle questionnaire (both self-completed by computer touchscreen and research nurse interview)[10].

Secondly, we used a subset of UKB participants living in Wales (comprising around half of the Welsh UKB population, n = 12,228), for whom linked primary and secondary care data were available via the Welsh Secure Anonymised Information Linkage databank (SAIL)[11] and the Patient Episode Database for Wales (PEDW) respectively.

Thirdly, we used a cohort of participants from the Clinical Practice Research Datalink (CPRD, n = 1,048,972). As linked primary and secondary care data were available for only half of Welsh UKB participants (capturing ~ 2% of all UKB participants),CPRD, a separate English dataset, was used to compare completeness of incident diabetes ascertainment using primary and secondary care data sources. CPRD contains anonymised primary care data for over 5 million patients from 650 general practices in England and Wales, with linked secondary care data from Hospital episode statistics (HES) for around 50%[12]. For both Welsh UKB and CPRD participants, primary care data are held in Read code format[13] and secondary care data in International Classification of Diseases (ICD) code format. While CPRD has the advantage of size, it is not linked to UKB (Fig 1).

Secondary care in-patient admission data linkage for England (HES), Scotland (Scottish Morbidity Record [SMR01]) and Wales (Patient Episode Database for Wales [PEDW]) is established in UKB. Linkage with English and Scottish primary care data is anticipated in the future, and it is envisaged this will be the chief source of incident diabetes diagnoses (Fig 1). The main source of information for prevalent diabetes at UKB inception is the baseline assessment. For incident cases occurring after recruitment, we have designed algorithms to interrogate primary and secondary care data, recognising that at least initially, researchers will have to rely on secondary care data alone, since primary care data linked to UKB are not yet available for the whole cohort.

A governing principle for UKB disease categorisation algorithms, given the focus on genetic analysis, is to ensure that cases are truly cases, whereas false negatives can be tolerated. Given the size of the dataset, this should only result in minimal contamination of controls.

UKB acquired ethics approval from the North West Multi-Centre Research Ethics Committee (06/MRE08/65). All investigations were conducted according to the principles in the Declaration of Helsinki. Written informed consent for data collection and record linkage was obtained from all UKB participants.

### Prevalence algorithms

#### Derivation

We designed algorithms for UKB baseline assessment data to assign presence and type of diabetes. This was achieved by combining clinical knowledge with multiple cross-tabulations of available data (comprising diagnosis, age at diagnosis, diabetes type, diabetes medications and diabetes complications). By examining patterns of congruent or contradictory evidence for diagnoses, we created logical rules capable of assigning or ruling out various diabetes-related ending states (S1 Appendix). The algorithms consisted of three stages: algorithm 1 assigned an overall likely diabetes status, algorithm 2 finalised type 1 diabetes diagnoses and algorithm 3 finalised type 2 diabetes diagnoses. As no algorithm can be definitive, we assigned a status of “probable” diabetes where there was greater certainty, and “possible" diabetes where there was less certainty.

#### Testing

a) **UKB baseline self-report data:** The algorithm was run on the baseline UKB dataset and final diabetes status recorded.
b) **UKB baseline self-report data vs. primary care data in the linked Welsh UKB sub-cohort:** Primary care data linked to UKB were available for a proportion of the Welsh participants. We ran the algorithm on this linked dataset. We then examined pertinent primary care data (diabetes-specific C10 diagnostic codes, diabetes medication, hyperglycaemia on blood testing and diabetes process of care codes e.g. for foot screening) against UKB final diabetes status to assess likely validity.
c) **UKB baseline self-report data vs. secondary care data in UKB:** Lastly, we compared final categorisations with prevalent diabetes diagnoses from secondary care data (HES) linked to UKB, i.e. those where the date of the first diabetes ICD-10 code (E10, E11, E13 or E14) in hospital admissions date preceded the UKB baseline assessment date.

#### Implementation

Prevalence algorithms were implemented in the UKB cohort at baseline to give a single estimation of prevalent diabetes status for all UKB participants.

### Incidence algorithms

#### Derivation

We designed algorithms for application to primary and secondary care data to establish incident diabetes cases. Our focus was on type 2 diabetes, given the age of UKB participants at recruitment. To assist generalisability to the UKB population, we restricted CPRD data to those on whom we had linked secondary care data, people aged 40–69 years on 1^st^ January 2006, (to reflect age entry criteria for UKB) Primary care algorithms were derived based on four types of evidence: 1) Diabetes diagnostic codes (considered separately as any diagnostic code and the more specific C10E [type 1 diabetes] or C10F [type 2 diabetes] codes, these are a requirement for the Quality Outcomes Framework [QOF] system[14]), 2) Diabetes medication, (excluding those on metformin only as this has other prescribing indications e.g. pre-diabetes, polycystic ovarian syndrome and is therefore not wholly diabetes specific), 3) Hyperglycaemia on blood results (defined as HbA_1c_≥6.5% or 48 mmol/mol, or fasting/ random/ unspecified glucose≥11.1 mmol/l) and 4) Presence of diabetes process of care codes (restricted to those routinely recorded for QOF monitoring purposes, e.g. retinopathy screening, foot checks etc.). The threshold for glucose was chosen because primary care records frequently do not specify whether glucose is fasting or not, and we wished to avoid false positives from a non-fasting glucose in the 7.0–11.1 mmol/l range. Using CPRD and the linked Welsh UKB sub-cohort, we used an iterative approach, cross-tabulating evidence at each step, to determine the logical steps to include in the algorithm and in what order. We then applied the final incidence algorithm to both databases. For CPRD, we excluded prevalent diabetes according to pre-existing C10 diabetes-specific Read codes, and for the Welsh dataset, we removed all those with prevalent diabetes according to our UKB algorithm.

When developing the incidence algorithms intended for secondary care data, we defined incident diabetes type based on ICD-10 codes (E10 = type 1 diabetes, E11 = type 2 diabetes, E13/E14 = unspecified diabetes). Prevalent diabetes was excluded as above.

For both primary and secondary care incidence algorithms, we derived event dates by taking the mid-point between the last primary care consultation/ hospital admission without diabetes and the date of the first diabetes Read code/ ICD code/ diabetes medication/ hyperglycaemic blood test/ fifth process of care code. If there were no previous consultations or admissions, we used the UK Biobank inception date. The date of the first diabetes Read code/ ICD code/ diabetes medication/ hyperglycaemic blood test/ fifth process of care code will be available to researchers separately if they wish to calculate the event date in an alternative manner.

#### Testing

a) **Choosing the most appropriate primary care codes in CPRD:** We compared the use of Read codes alone to establish incident diabetes in primary care with the combination of diabetes related measures (medication, hyperglycaemia and process of care codes). We performed a sensitivity analysis to establish whether we were missing many incident cases by excluding people in receipt of metformin alone with no other evidence of diabetes.
b) **Choosing the most appropriate number of secondary care codes in HES-linked CPRD:** Following a secondary care admission, a maximum of 20 distinct diagnostic codes can be recorded. We compared restriction of secondary care incident diabetes to the first two positions, versus mention in any position, with presence of diabetes Read codes (i.e. C10) in primary care.
c) **Primary vs. secondary care data in CPRD:** For individuals assigned incident diabetes status from primary care data, we compared sociodemographic characteristics, diabetes medication, blood tests, diabetes complications, cardiovascular disease (CVD) risk factors and co-morbidities in those with and without corroborating incident diagnoses in secondary care data. We also compared these characteristics in those for whom we only had a secondary care diagnosis of diabetes.
d) **Secondary care data in UKB:** Using our prevalence algorithm, we excluded participants with prevalent diabetes at baseline (classed as either “probable type 1 diabetes” or “probable type 2 diabetes”), then ran the secondary care data incidence algorithm in the UKB dataset and recorded final status.

#### Implementation

Incidence algorithms will be applied to the UKB cohort on a yearly basis, once the necessary linkages with primary care data have been made.

## Results

All 502,665 UKB participants were included, with a mean age of 57 years at baseline (2006–2010). Just over half (54%) were female and 95% of white European origin (Table 1).

**Table 1:** Baseline characteristics of UK Biobank (all), Welsh UK Biobank participants (with UK Biobank, primary and secondary care data linkage), and CPRD (primary and secondary care data linkage).

	UKB cohort	Linked Welsh UKB sub-cohort	CPRD
**N**	502, 665	12,228	1,101,101
**Female, %**	273,468 (54)	6,580 (54)	544,585 (50)
**Mean age, years**	57±8	53 ±8.2	53±8.5
**Ever smoked, %**	168,307 (34)	4,938 (44)	573,301 (52)
**Ethnic group, %**			
***European***	472,831 (95)	-	727,758 (66)
***South Asian***	8,067(2)	-	17,228 (2)
***African Caribbean***	8,066 (2)	-	10,645 (1)
***Other***	10,907 (2)	-	11,653 (1)
***Unknown***	-		333,817 (30)

There were 12,228 Welsh UKB participants with linked primary and secondary care data. In CPRD, records from 1,101,101 individuals who matched the age range at UKB recruitment and had linked primary and secondary care records were available. UKB participants’ smoking rates were much lower than CPRD participants’, suggesting the former are a healthier population. Around 5% of UKB individuals reported some form of diabetes, 4% reported receipt of anti-diabetic medication but only 0.3% reported microvascular diabetic complications (Table 2).

**Table 2:** Diabetes-related baseline self-report variables available in UK Biobank, N = 502,665.

***Touchscreen self-report data***		
Diagnoses	Diabetes diagnosed by doctor	26,408 (5.3)
	Gestational diabetes only	1,072 (0.4)
	Diabetes diagnosed by doctor (excluding gestational only)	25,336 (5.1)
	Age diabetes diagnosed, years	54 (46–60)
Medication	On insulin	5,613 (1.1)
	Started insulin within one year diagnosis of diabetes	3,034 (0.6)
***Nurse verbal interview***		
Diagnoses	Non-specified diabetes	21,738 (4.3)
	Age at non-specified diabetes diagnosis, years	55(47–61)
	Gestational diabetes	285 (0.10)
	Age at gestational diabetes diagnosis, years	37 (30–44)
	Type 1 diabetes	428 (0.09)
	Age at type 1 diabetes diagnosis, years	30 (20–43)
	Type 2 diabetes	3,367 (0.67)
	Age at type 2 diabetes diagnosis, years	56 (49–61)
Medications	Insulin	5,317 (20.0)
	Metformin	14,657 (55.5)
	Sulphonylureas	5,596 (21.1)
	Meglitinides	135 (0.51)
	Glitazones	1,997 (7.6)
	Any non-metformin oral anti-diabetic drug	6,809 (25.8)
	Any diabetes medication	19,045 (72.1)
Complications	Diabetic nephropathy	20 (0.08)
	Age at diabetic nephropathy, years	55 (40–63)
	Diabetic neuropathy/ ulcers	153 (0.6)
	Age at diabetic neuropathy, years	54 (46–60)
	Diabetic eye disease	1,172 (4.4)
	Age at diabetic eye disease, years	54 (46–61)
	Any diabetes complication	1,293 (4.9)

Data are n (%), n (% women) for gestational diabetes variables or median (IQR).

### Prevalence algorithms

#### Testing

**Fig 2:**
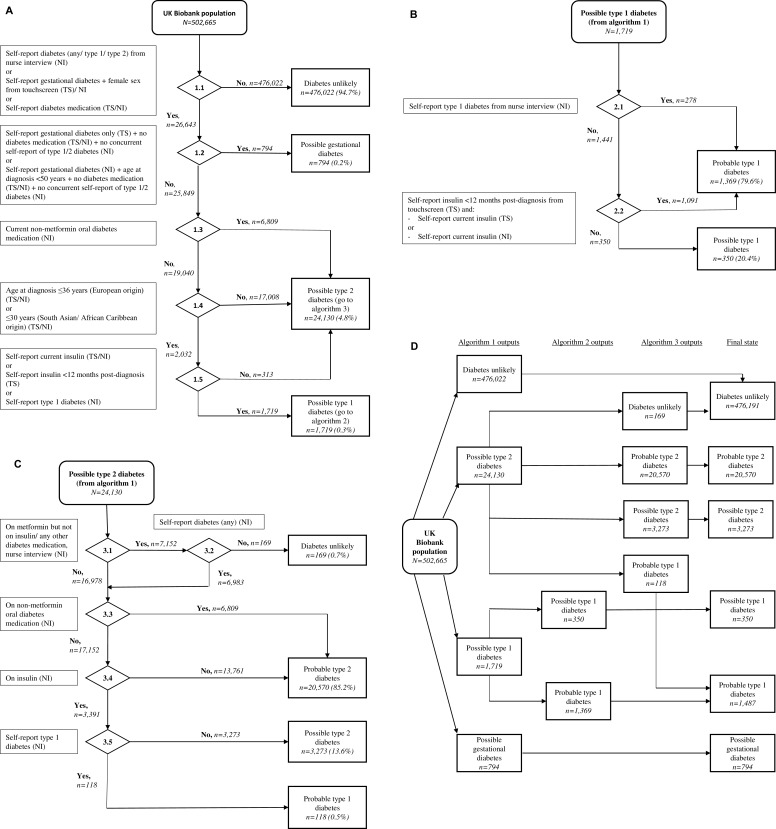
**(a) Prevalence algorithm 1: Distinction between diabetes presence or absence, and initial sorting of diabetes type using baseline UK Biobank assessment data.** See S1 appendix for rationale and further data for each step. **(b) Prevalence algorithm 2: Finalising type 1 diabetes diagnosis and classification into probable and possible categories.** See S1 appendix for rationale and further data for each step.**(c) Prevalence algorithm 3: Finalising type 2 diabetes diagnosis and classification into probable and possible categories.** See S1 appendix for rationale and further data for each step.**(d) Final diabetes diagnostic status in UKB. **

a) **UKB baseline self-report data:** After application of the algorithm, 95% (476,191/502,665) of UKB participants were deemed unlikely to have diabetes (Fig 2A–2D).

Proportions for “probable/ possible” prevalent type 1 diabetes and “probable/ possible” prevalent type 2 diabetes were 0.4% and 4.7% respectively. Similar proportions were found when the self-report algorithm was run on the Welsh UKB linked sub-cohort, though numbers in each category were relatively small (Table 3).

**Table 3:** Cross-tabulation of final diabetes status from UK Biobank prevalence algorithms against primary care diabetes data for linked Welsh UK Biobank participants.

Classification of diabetes status of UKB population according to UKB data		Diabetes unlikely	Uncertain diabetes status	Gestational diabetes	Probable type 1 diabetes	Possible type 1 diabetes	Probable type 2 diabetes	Possible type 2 diabetes
*Numbers (%)*, *N = 12*,*228*		11560(95)	0	23(0.2)	36(0.3)	9(0.1)	513(4.2)	87(0.7)
*Any diagnostic code for diabetes in primary care (N)*		10	0	2	29	8	386	70
*Diagnostic codes*	*Definite type 1 diabetes codes (C10E)*	0	0	0	22	2	3	15
	*Any type 1 diabetes code*^a^	0	0	0	27	3	5	21
	*Definite type 2 diabetes codes (C10F)*	9	0	1	6	2	378	55
	*Any type 2 diabetes code*^b^	9	0	1	7	4	383	58
	*Other/ non-specific diabetes codes*	3	0	0	20	4	163	43
	*Gestational codes*	0	0	2	0	0	0	1
	*Any type 1 diabetes code only*^c^	0	0	0	20	2	1	11
	*Any type 2 diabetes code only*^d^	9	0	0	0	3	379	48
	*Age at first diagnostic code*, *median (IQR)*	55 (44–61)		34 (27–39)	30 (23–38)	40 (33–46)	56 (50–60)	50 (43–55)
*Medication*	*Insulin*	1	0	0	28	4	49	62
	*Insulin only*	0	0	0	20	1	1	15
	*Metformin*	6	0	1	9	4	347	55
	*Metformin only*	3	0	1	1	0	97	2
	*Insulin and Metformin only*	0	0	0	8	1	1	12
	*Non-metformin oral anti-diabetic agent*	4	0	0	0	3	260	42
	*Insulin only or first*	0	0	0	28	2	1	24
*Other evidence*	*≥ 1 x HbA1c ≥6*.*5% or glucose≥11*.*1 mmol/l*	8	0	1	27	6	374	67
	*≥5 diabetes process of care codes*	7	0	1	22	6	356	63

Dates of relevant primary care codes precede the UK Biobank assessment date for each patient.

^a^Codes classified as Type 1 diabetes (definite), insulin dependent diabetes (probable type 1 diabetes) or juvenile onset diabetes (possible type 1 diabetes).

^b^Codes classified as Type 2 diabetes (definite), non-insulin dependent diabetes (probable type 2 diabetes) or adult onset diabetes (possible type 2 diabetes).

^c^Type 1 diabetes codes, no type 2 diabetes codes (there may be other or non-specific codes).

^d^Type 2 diabetes codes but no type 1 diabetes codes (there may be other or non-specific codes).

b) **UKB baseline self-report data vs. primary care data in the linked Welsh UKB sub-cohort:** Of the UKB Welsh primary care linked participants assigned "diabetes unlikely" status, only 0.001% (10/11,560) had evidence of diabetes in their primary care data (Table 3). Of those assigned "probable” type 1 diabetes status, 61% (22/36) had a definite C10E (type 1 specific) diagnostic code and 56% were on insulin exclusively (20/36). The median age at diagnosis in the probable type 1 diabetes group was 30 years. In contrast, of the 9 individuals assigned “possible” type 1 diabetes, 22% (2/9) had a C10E code in primary care, and only 11% (1/9) were on insulin only. Median age at diagnosis for this group was 40 years. Of those assigned "probable” type 2 diabetes status according to the UKB algorithms, 74% (378/513) had a C10F (type 2 specific) diagnostic code and an older median age at diagnosis (56 years). Only one participant was on insulin alone. With the less certain classification of “possible” type 2 diabetes, 63% (55/87) had a C10F code. Age at diagnosis was younger (50 years), and a greater proportion were on insulin (alone or in combination) than those classified as “probable” type 2 diabetes (71% versus 10%).
c) **UKB baseline self-report data vs. secondary care data in UKB:** In our comparison of prevalent diabetes status by self-report algorithm vs. secondary care data in UKB, we again found a very low occurrence (0.2%) of diabetes codes in secondary care data for participants assigned "diabetes unlikely" status from UKB questionnaire (Table 4).

**Table 4:** Comparison of final diabetes status from prevalence algorithms in UK Biobank versus diabetes diagnoses in secondary care data at baseline.

	Diabetes unlikely	Possible gestational diabetes	Probable type 1 diabetes	Possible type 1 diabetes	Probable type 2 diabetes	Possible type 2 diabetes
UK Biobank cohort (n = 502,665)	476,191	794	1,487	350	20,570	3,273
Hospital admissions codes present prior to baseline assessment date (% of all those in UKB)	284,780 (60)	539 (68)	1,163 (78)	289 (83)	14,659 (71)	2,606 (80)
Diabetes diagnosis in hospital admissions data at baseline in any position:						
*Any(% of all hospital data at baseline)*	642 (0.2)	13 (2)	963 (83)	242 (84)	6,430 (44)	1,866 (72)
*Type 1(% of diabetes diagnoses)*	39 (6)	1 (8)	734 (76)	98 (41)	115 (2)	508 (27)
*Type 2 (% of diabetes diagnoses)*	507 (80)	10 (77)	128 (14)	119 (49)	6,146 (96)	1,292 (69)
*Non-specific (% of diabetes diagnoses)*	120 (19)	2 (15)	100 (10)	23 (10)	491 (8)	175 (9)

Data are n (%). Derived from hospital admissions (available from 1997 onwards), date of first diabetes code used.

Of participants assigned "probable” type 1 diabetes, 83% had corroborative hospital admissions, of these, 76% had type 1-specific E10 diabetes diagnostic code. The corresponding figures for those with "probable” type 2 diabetes were 44% and 96% respectively. Diabetes type-specific hospital admission codes were less likely to occur in those with “possible” type 1 (41%) or “possible” type 2 diabetes (69%) with hospital admissions data.

#### Implementation

A range of final outcome categories was derived for the UKB baseline cohort, see above (Fig 1D).

### Incidence algorithms

#### Testing

**Fig 3:**
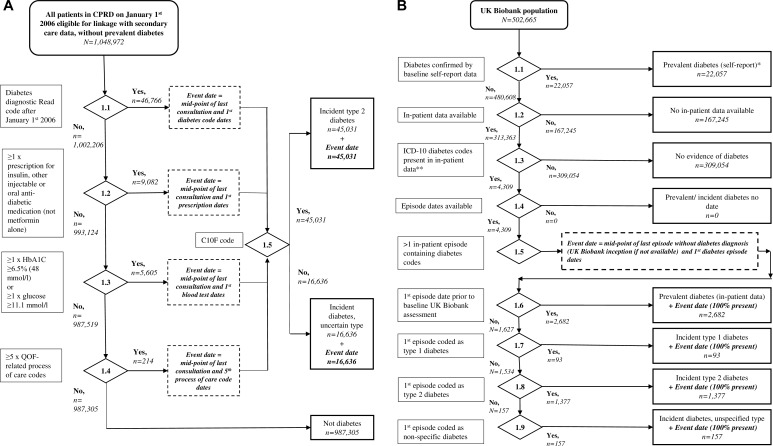
**a. Diabetes incidence algorithms for primary care data, run in CPRD**. **b. Diabetes incidence algorithm for secondary care data, run in UK Biobank-held in-patient data.** *Includes categories:probable type 1 diabetes, probable type 2 diabetes,. **ICD-10: E10, E11, E13, E14. Includes main or secondary diagnostic codes for in-patient data.

a) **Choosing the most appropriate primary care codes:** From the HES-linked CPRD dataset, restricted to the UKB age range at recruitment, out of 1,048,972 people without prevalent diabetes from either primary or secondary care data, 46,766 individuals had a first diabetes diagnostic Read code after 1^st^ January 2006 (Fig 2A). As data were drawn from primary care records, all diabetes diagnoses had dates; the ascertainment of these varied by criteria used (Fig 3A).

Of these, 95% had at least one corroborative item, such as a positive blood test, hypoglycaemic medication (excluding metformin alone), or ≥5 diabetes specific process of care codes in their primary care record. The majority (45,031, 96%), had a specific C10F diagnostic code, giving an incidence of type 2 diabetes over the 8 year period of follow up of 4%. An additional 16,636 individuals who did not have diabetes Read codes were identified from hypoglycaemic medication (excluding those on metformin only), blood tests or process of care codes, inflating the number of incident cases by 27%. These, along with individuals with a non-C10F diabetes-specific Read code were classified as incident diabetes of uncertain type. Excluding those with prevalent diabetes according to the UKB algorithm in the linked Welsh UKB dataset, 2.3% (265/11560) had de novo C10F Read codes, i.e. incident type 2 diabetes, during follow up. A sensitivity analysis of people in receipt of metformin alone (with no other evidence of diabetes) showed that, of a total of 19,447 individuals in CPRD (from 1^st^ January 2006, of the UKB age range and without prevalent diabetes) only 856 (4.4%) had no other evidence of incident diabetes in their primary care record (e.g. Read codes, hyperglycaemia or >5 diabetes process of care codes). Of the 987,305 people in CPRD deemed not to have incident diabetes (Fig 3A), there will thus be a maximum of 856 false negatives (0.09%).

b) **Choosing the most appropriate number of secondary care codes:** Of the 1,048,972 individuals in CPRD with no evidence of diabetes prior to January 2006, 25,959 had diabetes in positions 1–20, and 8,233 in positions 1–2 in their HES records after January 2006. Diabetes was identified in the primary care record, on the basis of a C10 code, in 74% (19,143) and 77% (6,373) of these individuals respectively. Since these proportions were similar, we performed the remainder of our comparison including all positions in the individual secondary care record. Using this definition, 2.5% (25,959) of the CPRD population had incident diabetes, according to secondary care records, over the follow-up period. Of the 25,959 total, 9% were initially diagnosed in 2006, 10% in 2007, 11% in 2008, 12% in 2009, 13% in 2010, and 14% in subsequent years to 2014.
c) **Primary vs. secondary care data in CPRD:** Of the 45,031 individuals in CPRD with incident diabetes in primary care, 18,440 (41%), had an incident code for diabetes in secondary care (Table 5). Compared to those with no secondary care code, those admitted with diabetes were older, more likely to be on medication, and more likely to suffer from co-morbidities. In contrast, of the 1,003,941 with no diabetes primary care Read code, 7,519 (0.007%) had secondary care evidence of diabetes. Unsurprisingly, these individuals had weak evidence of diabetes in their primary care record, such as medication use and hyperglycaemia on blood testing. They were also less likely to have microvascular, but not macrovascular complications. Thus using secondary care data alone, it is evident that 29% of incident cases appear to have little corroborative evidence of diabetes in primary care. Combining incident diabetes from secondary care with those detected in primary care would increase the incidence over the 8 year period of follow up from 4% to 5% in CPRD.

**Table 5:** Characteristics of those with incident diabetes from 1^st^ January 2006 to 1st January 2015, comparing those identified in primary care (with or without secondary care diagnosis) and those identified in secondary care alone in the CPRD database.

Denominator = 1,048,972 free from diabetes in CPRD on Jan 1^st^ 2006	Incident Type 2 diabetes diagnostic code (C10F) in primary care data (from Fig 3A) (n = 45,031)		No incident diabetes diagnostic code (C10F) in primary care data (n = 1,003,941)
	Incident Secondary care diabetes diagnostic code*	No secondary care diabetes diagnostic code at any time	Incident secondary care diabetes diagnostic code
**n**	18,440	26,591	7,519
**Age, years**	57 (8)	55 (8)	56 (8)
**Male sex**	10,795 (59)	15,796 (59)	4,318 (57)
**Insulin**	2,334 (13)	1,072 (4)	409 (5)
**Metformin**	13,908 (75)	17,613 (66)	496 (7)
**Other oral anti-diabetic agent**	7,437 (40)	6,675 (25)	253 (3)
**Any diabetes medication**	14,750 (80)	18,055 (68)	898 (12)
**≥ 1 HbA1c ≥ 6.5% (48 mmol/mol) or ≥ 1 blood glucose ≥ 11.1 mmol/l**	16,510 (90)	23,191 (88)	994 (22)
**≥5 process of care codes**	10,799 (59)	12,394 (47)	249 (3)
**Diabetes complications:**			
** • *Retinopathy***	3,697 (20)	3,951 (15)	170 (2)
** • *Neuropathy***	1,249 (7)	982 (4)	177 (2)
** • *Nephropathy (CKD Stages 3–5 coded)***	229 (1)	88 (0.3)	105 (1.4)
**BMI, kg/m**^**2**^	32 (6)	32 (6)	30 (6)
**Systolic blood pressure, mmHg**	142 (19)	142 (19)	138 (19)
**On medication for blood pressure**	14,812 (80)	19,119 (72)	4,020 (53)
**On statins**	15,639 (85)	20,980 (79)	3,103 (41)
**Co-morbidities:**			
** •*Coronary heart disease***	3,725 (20)	2,855 (11)	1,190 (16)
** •*Stroke***	139 (1)	81 (0.3)	36 (0.5)
** •*Heart failure***	1,019 (6)	683 (3)	298 (4)
** •*COPD/ asthma***	4,723 (26)	4,981 (19)	1,713 (23)

Data are mean±SD or n (%), extracted from primary care records on/ as close as possible after 1^st^ January 2006.

*Co-morbidities and diabetic complications are at any time.

d) **Secondary care data in UKB:** Using hospital admissions data linked to UKB identified 1,627 incident cases of diabetes (Fig 2B), equating to around 0.3% incidence since inception (2006–2010 to end of 2012 with complete data to end 2010), about 20% of that anticipated from CPRD data using the same definition. All cases had event dates specified in hospital records for this run of the algorithm, though in future instances if event dates are not present, cases will not be classed as incident, since it will not be possible to distinguish between incident and prevalent cases. Most (85%) cases were categorised as type 2 diabetes.

#### Implementation

**Fig 4:**
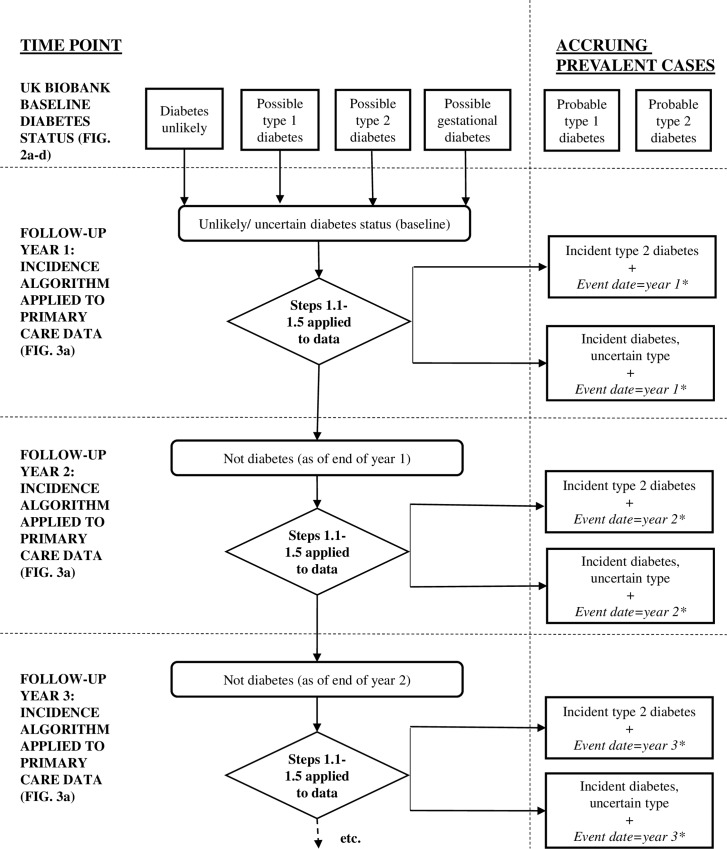
Flow of participants identified with diabetes in UK Biobank. *or mid-point of last consultation/episode without diabetes diagnosis (UK Biobank inception if not available) and 1^st^ diabetes diagnosis dates.

It is envisaged that algorithms determining incidence diabetes will be applied to the UKB cohort on a yearly basis, once appropriate primary care data become available (Fig 4).

Each time the algorithms are run, the previous years’ incident cases will move to the pool of prevalent cases, with the date of onset being provided by the algorithm in parallel with the diagnosis. Incidence algorithms will be applied to those with a “diabetes unlikely”, “possible type 1 diabetes”, “possible type 2 diabetes” and “gestational diabetes” alike, since the latter three states still constitute clinical uncertainty. Therefore event dates for diabetes diagnoses will be calculated (see “incidence algorithms; derivation” section above for method) using the point where diabetes is positively confirmed. This algorithm will focus on incident type 2 diabetes; given the age range of the UKB cohort at baseline (37–73 years), incident diagnoses of gestational or type 1 diabetes will be rare.

## Discussion

We have described the development and testing of algorithms to identify prevalent and incident diabetes case status for participants in the UKB cohort, using self-report, primary and secondary care data. The algorithms will be implemented at source and their results made available to researchers on request. Our prevalence algorithm for self-report data makes a clear distinction on the basis of certainty between “probable” and “possible” cases of each type of diabetes. Probable cases of type 1 and 2 diabetes have strong corroborative data in primary care. Such corroboration is weaker for possible cases of type 1 and type 2 diabetes. Use of primary care diagnostic Read codes alone appears valid in ascertaining incident type 2 diabetes. Using secondary care data, yield of incident diabetes cases is increased 3-fold if any mention of diabetes in any one of 20 positions is used, versus restriction to the first two positions, without appreciable loss of validity, when compared to primary care data. A significant proportion of people with incident diabetes (14%) are detectable in secondary care data alone.

Results of the prevalence algorithms based on self-report data showed proportions of overall type 1 diabetes (~0.5%) and type 2 diabetes (~5%) in UKB being similar to those reported in the general population[15]. For those designated as “diabetes unlikely”, virtually no participants had diabetes codes in primary (0.001%) or secondary (0.2%) care records. The majority of those with a “probable” type 1 diabetes diagnosis had a diabetes code in both primary and in secondary care, though proportions for the more specific type 1 diabetes codes were lower. Diagnostic confusion and misclassification for type 1 diabetes has been highlighted[8,9]. Whilst proportions with any diabetes code in hospital admissions data were similar in those with “possible”, as opposed to those with “probable” type 1 diabetes (84% and 83% respectively), a specific type 1 code was only present in 41% of “possible”, versus 76% in those with a “probable” diagnosis. For type 2 diabetes, while proportions with type 2 diabetes specific (C10F) Read code in primary care were similarly high in those with a “probable” versus “possible” diagnosis (74% versus 63%), the latter were younger at diagnosis, and more likely to be on insulin alone. In support, 27% of those with hospital admission data assigned to “possible” type 2 diabetes had a type 1 diabetes specific ICD code, compared to just 2% of those with “probable” type 2 diabetes. These findings, of greater use of insulin alone and a greater likelihood of having type 1 diabetes specific ICD codes, in people assigned “possible” rather than “probable” diabetes suggests that this category includes a significant proportion of people who have type 1 diabetes (with an older age of onset), and people with latent autoimmune diabetes in adults (LADA).

For incident diabetes, we derived a primary care algorithm that included only those with a type 2 diabetes-specific Read code, the majority of whom had at least one additional confirmatory piece of evidence from medication, hyperglycaemia and diabetes specific process of care codes. This provides reassurance that these individuals have type 2 diabetes. The remainder, who did not have a specific type 2 diabetes Read code, or who only had other primary care evidence of diabetes (e.g. medications), but no diabetes Read code, were classified as “uncertain”. This distinction allows researchers to choose how strict their definition of diabetes should be; for those who require greater precision, we would recommend adhering to the type 2 diabetes specific Read code diagnosis alone. The CPRD analysis used a previous C10F Read code to remove prevalent diabetes and yielded incidence rates of diabetes of 4%, whereas analysies in the linked Welsh UKB dataset, which removed those with prevalent diabetes according to our UKB algorithm, yielded a lower incidence of 2.3%. This discrepancy is likely due to historical under-coding of diabetes in primary care, missing around 24% of prevalent cases according to our analysis of the linked Welsh UKB dataset. With incentives to improve coding in the last decade, individuals with long-standing diabetes may have been given a recent diagnostic Read code, artificially inflating incidence in CPRD. Using the UKB-specific prevalence algorithm at baseline should deal with potential misclassification of prevalent as incident cases in UKB.

Using secondary care admission data, we show that diabetes is not the primary or secondary cause for hospital admission in the majority of cases. A similar proportion of individuals possessed diabetes-specific C10F codes, regardless of whether the diabetes ICD-10 code on admission occupied either the first or second coded position versus any mention of diabetes on admission, supporting our decision to include all those in secondary care with any mention of diabetes. Of those identified in primary care, just under half had diabetes recorded on hospital admission data. These individuals were older, more likely to be on insulin, and more likely to have diabetes complications than those without secondary care evidence of diabetes, as anticipated. A proportion of individuals were identified with type 2 diabetes from secondary care records alone; unsurprisingly these individuals had less evidence of medication, hyperglycaemia and microvascular (but not macrovascular) complications in their primary care record than those identified with the C10F code in primary care. Combining both primary and secondary care data to identify diabetes, 86% of the total would be identifiable in primary care, (with secondary care evidence of diabetes in 41% of these), and 14% of the total would be identified in secondary care alone. Notably, secondary care out-patient data does not carry ICD codes and cannot be used for capturing cases.

In CPRD, over the 8 year period of follow up from 2006, using primary care as the only source would yield a diabetes incidence of 4%, secondary care alone 2.5%, and in combination 5%. The limited evidence of diabetes in the primary care records of those identified through secondary care alone, makes the use of secondary care data alone, or combining those with secondary care evidence of diabetes alone with those with primary care evidence difficult. In the absence of primary care data, researchers using secondary care identification of diabetes will need to recognise that nearly a third have no evidence of diabetes in primary care, and appear different, in terms of risk factors and microvascular complications, to those identified in primary care. A similar argument applies to combining those found in secondary care alone with those found in primary care.

Our analyses of secondary care data in UKB provided a much lower yield of incident cases than anticipated from our CPRD analysis. We would have anticipated around 8000 incident cases based on CPRD, whereas only just over a 1600 were found. The follow up for CPRD commenced in 2006, while that for UKB spans 2006–2010, due to staggered recruitment. In addition, while CPRD has linked HES data to March 2014, that for UKB is linked to only 2012, and complete only to December 2010. In addition, as stated above, due to historical undercoding of diabetes in primary care, it is likely that we excluded only 76% of all prevalent cases in CPRD, thereby inflating incidence in secondary care. We estimated that, allowing for the impact of differences in follow up and definition of prevalent diabetes between UKB and CPRD datasets, the number of anticipated incident cases would be reduced by around 75%, i.e. a yield of 2000 cases, still higher that actually observed in UKB. With a 5.5% response rate, UKB participants are not representative of the general population, unlike CPRD registrants. While prevalence of type 1 and type 2 diabetes in UKB are as anticipated from population studies, more generally, UKB participants are clearly healthier than their general population counterparts, as demonstrated by their much lower smoking rates. We hypothesise that participation in UKB attracted distinct groups of individuals, those with established disease on the one hand, and the very healthy on the other. Thus whilst prevalence of diabetes may not differ from population estimates, it is only the pool of healthy participants who can contribute to incidence, accounting for the much lower rates of incident disease compared to population samples. Our data underline the dangers of using UKB data to estimate disease burden (prevalence or incidence) and should not be used for this purpose[10]. However, we do not have evidence to suggest that risk factor associations with diabetes differ markedly between UKB participants and the general population.

Event dates for our incidence algorithms were derived by taking the mid-point between the last primary care consultation or hospital admission and the date of the first diabetes code. This method is likely to give an event date closer to the true onset of diabetes than the date of the first diabetes code alone (though the latter will be made available to researchers), and will prevent artefacts in time-to-event analyses, e.g. appearance of incident diabetes being associated with immediate myocardial infarction. However we acknowledge this approach is a compromise, and that the true date of onset cannot be established with the available data.

The chief limitation to this work is data quality–others have consistently shown errors in self-report, and primary and secondary care data recording[4,5,7–9]. Whilst the principle aim of our work was to overcome these inaccuracies, all algorithms were obligatorily data-driven, with no gold standard for comparison. Another drawback is a potential delay in ascertainment of incident cases resulting from the algorithm’s exclusion of individuals receiving metformin alone, who are likely to be either newly-diagnosed or well-controlled. However, a sensitivity analysis indicated that, of the 987,305 people in CPRD deemed not to have incident diabetes, even in the most adverse scenario (where all the people on metformin alone with no other evidence of diabetes genuinely have diabetes), there would be a maximum of 856 (0.09%) false negatives. Furthermore, we were willing to tolerate these potential false negatives to avoid false positive classification of people receiving metformin for pre-diabetes, obesity or polycystic ovarian syndrome.

We conclude that use of baseline self-report diabetes data is a pragmatic and valid approach to defining prevalent cases of diabetes, without missing cases known to primary or secondary care, and appears to rightly distinguish between “probable” and “possible” states. Regarding incidence, we suggest that restriction to the type 2 diabetes specific Read code (C10F) in primary care has the greatest precision. Secondary care data captures around half of all cases with diabetes, with 70% having corroborative evidence of diabetes in their primary care record, in the form of a specific diagnostic Read code. We would caution against using diabetes-specific information, in the form of medication or blood tests, in the absence of these Read codes, or combining individuals identified in secondary care alone with those identified in primary care. Whilst we make recommendations, it is acknowledged that UKB data will be used to address multiple questions, with varying levels of precision, and tolerance of false negatives. We have therefore, where possible, categorised groups with differing levels of diagnostic certainty, allowing investigators themselves to choose which categories to use in their analyses.

## Supporting Information

S1 Appendix Rationale for logical rules in prevalence algorithms used on UK Biobank recruitment questionnaire data. TS = participants entered data themselves via electronic touch screen, NI = data accrued from individual nurse interview, GDM = gestational diabetes mellitus.
(DOCX)
