# Supplementary material for: Algorithms for the Capture and Adjudication of Prevalent and Incident Diabetes in UK Biobank
Source: PLoS One. 2016 Sep 15;11(9):e0162388. doi: 10.1371/journal.pone.0162388 (PMC5025160; doi:10.1371/journal.pone.0162388)
Supplement: S1 Appendix — TS = participants entered data themselves via electronic touch screen, NI = data accrued from individual nurse interview, GDM = gestational diabetes mellitus. (DOCX) [file pone.0162388.s001.docx]

**S1 Appendix. Rationale for logical rules in prevalence algorithms used on UK Biobank recruitment questionnaire data.** TS=participants entered data themselves via electronic touch screen, NI=data accrued from individual nurse interview, GDM=gestational diabetes mellitus.

| **Rule** | **Purpose** | **Rationale** |
| --- | --- | --- |
| **Prevalence algorithm 1 – Distinguishes between diabetes presence/absence** | | |
| **1.1** | Identifies participants in whom diabetes unlikely | Based on absence of self-reported diabetes diagnoses or medication. The exception was diabetes (non-GDM) report by TS alone (n=1,025), which was not used to indicate possible diabetes, since only 3% (36/1,025) reported other markers of diabetes, compared with 73% (18,572/25,383) of those with diabetes diagnoses by TS and NI, and 16% (35/217) of those with NI diagnoses alone.  Presence of diabetes complications was not used to rule in/ out cases, due to low prevalence (0.3%). |
| **1.2** | Identifies possible GDM only | Based on TS or NI self-report of GDM. Participants excluded at this step did not have any evidence of current diabetes (medication or self-report of other types of diabetes), i.e. those who may have had pre -existing diabetes or developed type 1 or 2 diabetes subsequent to GDM continue in the algorithm. This included a total of 338/1072 (32%) women reporting GDM by TS and 41/234 (18%) by NI with evidence of current diabetes who were not currently pregnant. In addition, 22 women who reported age at GDM diagnosis of ≥50 years also continued in the algorithm (i.e. were **not** assigned possible GDM status). |
| **1.3** | Identifies possible type 2 diabetes from use of non-metformin oral anti-diabetic drugs | Use of this type of medication likely to be exclusive to type 2 diabetes. |
| **1.4** | Identifies possible type 2 diabetes from ethnicity-specific age at diagnosis | Age of onset of diabetes is younger in the UK’s largest ethnic minority groups; South Asians and African Caribbeans. We applied a priori-determined ethnic-specific cut-points to define older vs. younger age at diagnosis – indicating higher likelihood of type 2 vs. type 1 diagnoses respectively. Age of onset was taken from NI data if NI and TS data were available, and taken from TS data if NI data were not available, 1243 participants with no age data went into the "possible type 2 diabetes" arm. |
| **1.5** | Identifies possible type 1 diabetes from either insulin use (TS or NI), insulin commencement within 1 year of diagnosis, or self-report of type 1 diabetes (NI) | Almost all participants with type 1 diabetes will be on insulin; coupled with a lack of non-metformin oral anti-diabetic drugs and a younger age at onset (see rules 1.4 and 1.5), specificity for type 1 diabetes is increased.  Immediate commencement of insulin after diagnosis is obligatory for type 1diabetes. We included the criterion of commencement of insulin at less than 12 months post-diagnosis in addition to current insulin use as 96 people entering rule 1.6 reported insulin use at less than 12 months but no current insulin. These people are then carried forward to the next flowchart for finalising type 1 diabetes status, and are labelled as "possible type 1 diabetes". Otherwise they would be excluded, but it is possible to have been on insulin initially and not currently, e.g. due to pancreatic transplant/ incorrect diagnosis.  Characteristics of participants self-reporting (NI) type 1 diabetes at this stage in the algorithm appear to be congruent with the diagnosis – their average age at diagnosis was 21±9 years, 98% (TS, 273/278) or 95% (NI, 265/278) were on current insulin, only 10% (30/278) were on metformin and 92% (194/211) started insulin within 1 year of diagnosis. However, due to the low number of participants specifying this diagnosis at NI overall 0.09% (428/502,665), we sought other evidence of type 1 diabetes in addition. NB: there was no option to specify diabetes type on TS self-report. |
| **Prevalence algorithm 2. Interrogates type 1 diabetes categorisation and distinguishes probable from possible** | | |
| **2.1** | Identifies probable type 1 diabetes from self-report of type 1 diabetes, nurse interview | Deemed to indicate probable type 1 diabetes – see above. NB - 10 participants co-reported type 1 and type 2 diabetes at NI, these were re-classified as non-specific diabetes. |
| **2.2** | Identifies probable type 1 diabetes from insulin commencement within 1 year of diagnosis and current insulin use. | Of those 1441 entering this algorithm without nurse report of type 1 diabetes, 1121 (78%) received insulin within a year of diagnosis, 1394 (97%) self reported current insulin use on touchscreen, and 1373 (95%) reported current insulin use to the nurse. Metformin use was reported in 52% of those with “possible” versus 11% of those with “probable” type 1 diabetes. Similarly, proportions co-reporting type 2 diabetes were 9% in people with “possible” versus 0.5% in people with “probable” type 1 diabetes. |
| **Prevalence algorithm 3. Interrogates type 2 diabetes categorisation and distinguishes probable from possible** | | |
| **3.1** | Identifies participants whose only diabetic medication is metformin | Metformin can be used to treat conditions other than diabetes, e.g. polycystic ovarian syndrome, therefore treatment with metformin only is less specific for diabetes than if used in conjunction with other anti-diabetic drugs. |
| **3.2** | Identifies participants whose only diabetic medication is metformin but did not report diabetes at nurse interview | With the exception of 12/169 (7%) who reported diabetes on TS, no other diabetes self-report data was present for participants who were deemed unlikely to have diabetes at this stage. |
| **3.3** | Identifies participants who are on oral anti-diabetic agents other than metformin (i.e. likely to be specific for type 2 diabetes) | Participants assigned "probable type 2 diabetes" status at this stage were more likely than those who continued in the algorithm to have developed diabetes at an older age (55±9 vs. 52±9 years, p<0.001) and to be taking metformin concurrently (79%, 5404/6809 vs. 51%, 8750/17152, p<0.001), and less likely to be on insulin (8%, 546/6809 vs. 51%, 8750/17152, p<0.001). |
| **3.4** | Identifies participants not on insulin (i.e. unlikely to have type 1 diabetes) | Participants who were assigned "probable type 2 diabetes" status (i.e. were not receiving insulin) at this stage were more likely than those who continued in the algorithm to have developed diabetes at an older age (56±9 vs. 46±9 years, p<0.001), though rates of metformin use were similar (51%, 6983/13761 vs. 52%, 1767/3391, p=0.2). |
| **3.5** | Identifies probable type 1 diabetes on the basis of self-report of type 1 diabetes, nurse interview | Participants who were assigned "probable type 1 diabetes" status at this stage were more likely than those who were assigned "possible type 2 diabetes" status to have developed diabetes at a younger age (48±8 vs. 50±7 years, p<0.001) and to have commenced insulin at less than one year after diagnosis (72%, n=79/109 vs. 39%, n=1197/3040, p<0.001), and less likely to be taking metformin currently (27%, n=32/118 vs. 53%, n=1735/3273, p<0.001).  Participants were assigned "possible type 2 diabetes" rather than "probable type 2 diabetes" status at this stage as insulin use and relatively high rates of commencement of insulin within a year of diagnosis (39%, n=1197/3040) cast doubt over a type 2 diagnosis. |
